# Environmental scan of mobile apps for promoting sexual and reproductive health of adolescents in low- and middle-income countries

**DOI:** 10.3389/fpubh.2022.993795

**Published:** 2022-11-23

**Authors:** Aneri Patel, Samantha Louie-Poon, Samar Kauser, Zohra Lassi, Salima Meherali

**Affiliations:** Child Development, Faculty of Nursing, University of Alberta, Edmonton, AB, Canada

**Keywords:** sex education, adolescents, sexual and reproductive health education, mobile applications, mHealth

## Abstract

**Background:**

Adolescence is a period of emotional, mental, and physical change. To increase health seeking behaviors, reduce risky sexual behavior, and improve sexual and reproductive health (SRH) knowledge, adolescents require support and access to SRH services. Providing evidence-informed SRH knowledge to adolescents in low- and middle-income countries (LMICs) can be a challenge as they face unique barriers such as lack of confidentiality, fear of refusal, and stigma from cultural norms. Increasing availability of mobile apps necessitates a comprehensive evaluation of the quality and classification of these SRH mobile applications so that accurate and evidence-based information is reaching its users. Failure to provide SRH services can have damaging effects throughout their development.

**Objective:**

Provide an overview of current adolescent SRH (ASRH) mobile applications targeting adolescents in LMICs by evaluating their quality and classifying their characteristics.

**Methods:**

21 search terms related to ASRH mobile apps was developed. These terms were searched in the Apple IOS store and Google Play stores. Inclusion and exclusion criteria were used to screen these apps. Resulting apps were assessed using the Mobile App Rating Scale (MARS) tool. Data extracted was used to rank order each app and identify any gaps in quality.

**Results:**

Search strategy yielded 2,165 mobile apps. Of these, only 8 were assessed using the MARS tool. Functionality subdomain scored highest at 4.6, while Information scored lowest at 2.5. None of the assessed apps contained information on the MARS items: Evidence base and Goals. Too Shy to Ask had the highest individual app mean score of 4.1, while e-SRHR scored lowest at 2.3.

**Conclusions:**

The goal of this study is to classify and rate the quality of mobile apps designed to promote ASRH behaviors and knowledge in LMICs. Numerous apps were reviewed and all of them failed to provide evidence-based and goal oriented SRH information. Strengths include ease of use, navigation, and gestural designs. Weaknesses include evidence base, goals, willingness to pay, customization, and interactivity. These findings can be potentially used to guide future app development and educate decision makers responsible for policy changes.

## Introduction

Adolescence is a dynamic process where an individual goes through many physical and psychosocial changes. In this phase, individuals are transitioning from childhood to adulthood by going through rapid changes in sexual, emotional, social, and mental health (1). The World Health Organization (WHO) defines adolescents as persons between the ages of 10–19 (2). This is a vulnerable time as they must learn to navigate a variety of obstacles ranging from sexual experimentation, developing sexual identities, sexual relations, and lack of self-esteem that may impact their physical, mental, and emotional health. Furthermore, insufficient knowledge of sexual and reproductive health (SRH) in adolescents can contribute to unintended pregnancies, unsafe abortions, and sexually transmitted infections (STIs) (3). Globally, over seven million adolescent females unintentionally become pregnant as a result of poor SRH education (4). While many developed and higher-income countries have legalized abortion, many developing and lower-income countries still contain repressive laws on abortion (5). In fact, of the estimated 20 million global unsafe abortions, 95% of them occur in developing countries (5). A large gap exists in SRH between higher income and lower-and middle-income countries (LMICs). For instance, maternal morbidity in Sub-Saharan Africa is 1 in 13 while in the United States it is 1 in 2000 (6). This substantial difference in higher-income countries compared to LMICs necessitates greater support for adolescents in these regions regarding increasing health-seeking behavior, and accessibility to SRH services and information such as birth control, STI prevention, and LGBTQ+ information. However, adolescents in LMIC seeking SRH services and information often find that their needs are unmet and experience unique barriers to accessing SRH information and services (7). Some of these include misconceptions regarding SRH information due to familial influences such as low parental education and low family socioeconomic status (8), accessing SRH services and information is considered a stigma and taboo for young unmarried people, hesitations surrounding healthcare provider confidentiality (7) and fear of refusal also exists among many adolescents as being sexually active at a young, unmarried age goes against many societal and cultural norms in LMICs (9). For example, menstruation is greatly neglected in LMICs as many girls have misconceptions regarding menstruation resulting in feelings of unpreparedness, fear, and anxiety (10). Although adolescents have physically developed bodies, their growing cognitive and emotional faculties necessitate further educational support on SRH. Knowledge and safe practices gained by adolescents from SRH services will support and guide their health and developmental milestones before they transition into adulthood. Failing to provide confidential, accessible, and stigma-free SRH information and services to adolescents irrespective of country income status can be detrimental to their current and future relationships, sexual health, and overall well-being (11).

Increasing production of mobile phones and the availability of affordable data plans in LMICs are steadily transitioning these countries more into the digital world (12). This can be attributed to the increased enthusiasm adolescents display about new technologies (13). For instance, 70% of South African adolescents own mobile phones and 6% use Internet services daily (14). As a result of increasing mobile ownership, digital tools for promoting adolescent SRH have achieved many positive results such as increased use of condoms and awareness of the negative consequences that arise from risky sexual behaviors (15). Using mobile apps to promote SRH education and services can be highly effective in overcoming the barriers that prevent adolescents in LMICs from accessing health services free from stigma and lack of privacy. Another appeal of mobile technology as a health promotion tool is that it can provide accurate, cost-effective, confidential, and tailored health promotion information to adolescents (9). A study in Ghana found that mobile health (mHealth) programs for adolescent girls were effective in increasing SRH knowledge and parental support despite the cultural barriers present in the target population (16). Interactive components of mobile technologies report improved adherence, involvement, and motivation among adolescents learning safe sex practices (17). Previous interventions regarding mobile technologies illustrate that mobile phone applications particularly have great potential to provide safe, accurate, and high-quality SRH information and support to adolescents in LMICs (9). Mhealth interventions contain a broad range of technologies, such as websites, web applications, and mobile applications to name a few. Unlike other alternative digital sources, mobile applications allow users to interact offline and provide a greater sense of security as apps must first be approved by the app stores before use (18). On the other hand, web applications require an active internet connection to function (19). Mobile application use is thus more valuable in LMICs as resource constraints often struggle to provide quality, high-speed internet (20). For these reasons, only mobile applications were investigated in this study.

With the increase in cell phone accessibility and usage in LMICs, access to SRH knowledge is easier for adolescents (21). However, little information is available about the quality and characteristics of these mobile apps. A systematic approach is needed to evaluate the quality of adolescents' SRH mobile apps to ensure that adolescents are receiving the most accurate information in an accessible manner. Mobile apps that are culturally conscious and inclusive are more effective in improving sexual health practices and outcomes (22). Adolescents need to receive proper evidence-based SRH information to make informed decisions. Therefore, evaluating the quality and characteristics of mobile apps developed for adolescents living in LMICs is imperative to improve ASRH outcomes.

In this study, a systemic evaluation of the existing SRH mobile apps developed specifically for the adolescent population in LMICs will be achieved through an environmental scan approach. While previous studies have been done to evaluate the quality of ASRH mobile apps, few have been focused on LMICs. For instance, a content analysis on comprehensive sexual education apps focused on teenagers and young adults in the United States concluded that current apps mostly contain education on STI and pregnancy prevention rather than a holistic and comprehensive based education (23). Content such as anatomy and physiology, pregnancy and reproduction, personal safety, healthy relationships, identity, sexual pleasure, STIs and HIV, and communication and interpersonal skills were analyzed (23). Despite these categories, app quality and characteristics such as the presence of evidence-based information, ease of use, and appropriateness for the target group was not evaluated. Furthermore, this study also highlights the need for comprehensive SRH for adolescents in LMICs where reproductive outcomes and socioeconomic statuses tend to be poorer (23). Another example of a lack of ASRH in LMICs can be seen in a mobile app named GirlTalk which was developed by researchers to educate adolescent girls regarding sexual and reproductive health in the United States or more specifically Rhode Island (24). Although this study concluded that Girl Talk is a reliable and accessible educational tool for adolescent girls to use, there is a need for such studies to expand to adolescents of all genders in LMICs (24).

As opposed to previous research, this study will systematically classify and rate the quality of SRH mobile apps for adolescents residing in LMICs. The findings from the environmental scan will offer information on [1] the quality and characteristics of mobile apps available to promote adolescent SRH in LMICs and, [2] areas of improvement for the development of future apps, thereby potentially improving access to SRH information to adolescents in LMICs.

## Methods

### Study designs

An environmental scan (ES) is used to identify current SRH mobile applications (hereinafter referred to as “apps”) available in the IOS App store and Android Play store. Furthermore, the Mobile Application Rating Scale (MARS) screening assessment tool is used to perform app quality assessments. Although environmental scans are commonly used in the business sector, they are not as well-established in the healthcare services context (25). A working definition developed by researchers of an ES scoping review defined environmental scans as a form of inquiry that collects and synthesizes existing information from internal or external environments to examine current landscapes, practices, policies, etc. so that future policies, models, and structures can be built to improve patient safety, programs, and overall quality (26). An ES is particularly advantageous as it allows for information to be gathered from an environment where evidence-based information surrounding a specified topic has not been developed yet (27). In comparison to rapid reviews and other forms of literature reviews, collecting information for an ES is not restricted to databases containing peer-reviewed and or grey literature. For this reason, an ES was the preferred method of research due to the lack of peer-reviewed and grey literature publications present that assessed the quality and characteristics of mobile apps currently available. An ES is also beneficial in that it allows us to systematically assess internal factors that may be impacting the quality and characteristics of ASRH mobile apps in LMICs (28). Internal factors are application quality and its characteristics. Through assessment of internal derived factors, all data trends that impact the quality of mobile apps can be used as evidence to educate decision-makers and guide app developers on improvements needed for ASRH in LMICs (29). After mobile apps are scanned from the app stores, the Mobile Application Rating Scale (MARS) screening assessment tool is used to perform app quality assessment.

### Search strategy

Since our research objective is to assess SRH apps for adolescents in LMICs, we searched for apps available in the largest stores in the world: Apple IOS store and Google Play store. According to the website Statista, the Google Play store contains the largest number of available apps in the market at around 3.5 million smartphone applications (30). Apple store is the second largest app store in the world and contains roughly 2.2 million apps available to the public (30). As there were limited to no studies investigating mobile apps for ASRH in LMICs in peer-reviewed and grey literature, information on these apps could not be collected from these sources. For this reason, mobile apps were scanned from the App store and Play store. To discern whether the mobile apps collected focused on ASRH in LMICs, a set of inclusion and exclusion criteria were created to screen the mobile apps. Before searching for the apps, 21 key search terms were created and run through the custom software individually. This custom python 3-based software was built using an iTunes App store Scraper and a Play store Scraper, in which a CSV file of non-personalized search results was generated. This was used to create an index of applications and their characteristics. The databases of apps searched were the Google Play Store and Apple App Store, from both the US and Canada. The custom software returned the first 50 search results for each search term, for each of the 4 stores searched, before removing duplicate results and creating two final lists of apps: one from the CA and US Play Store, and one from the CA and US App Store. Apps from these stores were then collected and categorised based on app title, publisher, description, primary category, and price. 21 key search terms that were developed and then searched for in our custom electronic software:

1. Adolescents
2. Teenagers
3. Youth
4. Sex
5. Sexuality
6. Sexual health
7. Reproductive health
8. Sexual and reproductive health education
9. Sexual and reproductive health services
10. Pregnancy
11. Contraceptives
12. Safe Sex Practices
13. Sexually transmitted diseases (STDs)
14. Sexually transmitted infections (STIs)
15. Abortion
16. Low- and Middle-Income Countries
17. Developing Countries
18. Mobile Applications
19. E-Health Literacy
20. Mobile Health (mHealth)
21. Pre-marital sex

### Application screening

Our search strategy yielded a total of 1107 results for the App store and 1060 for the Play store. These results were organized into a Microsoft Excel 2019 spreadsheet in tabular format and were screened based on our inclusion and exclusion criteria. Microsoft Excel was used given the ability of this software to permit a systematic screening process in an organized manner. For an app to be considered, it must meet all inclusion criteria and exclusion criteria. To assess whether an app met the criteria, reviewers examined the app name, description, pictures, and developer name. For example, when discerning if an app targeted LMIC adolescents, reviewers examined the “About this App” section for any indications of the name of any LMICs or words such as “teenager,” “adolescents, and “youth.” One reviewer screened apps against our a-priori inclusion and exclusion criteria, which was verified for accuracy and completeness by a second independent reviewer. Any differences in app screening were discussed between reviewers until a consensus was reached.

Five inclusion criteria and four exclusion criteria were:

Inclusion criteria:

1. Contains content related to sexual health education
2. App's intended audience is adolescents (age 10-19 years)
3. App still exists in the Play/App store when being assessed.
4. Target to adolescents living in LMICs as defined by World Bank
5. Compatible for phones available to LMICs

Exclusion criteria:

1. Did not address any component of sexual health/sexuality
2. Not in English
3. Paid
4. Developed for a specific event such as a conference

Before apps can move onto the assessment phase, we ensured that apps must target the population of interest and contain any component of sexual health education. Specifically, they target adolescents living in LMICs so that the desired demographic can be investigated. In addition to demographic, apps must still exist in the stores when being assessed so that reviewers can evaluate them. Lastly, the app must be compatible with phones available in LMICs so that it can still be used by people in those regions and reviewers can provide an accurate overview of current apps available in the market. If an app did not address any component of sexual and reproductive health, it was excluded from the study to maintain objectivity. Apps that did not contain content available in English did not proceed into the assessment phase since the research team's primary language is English and thus cannot conduct assessments unless the content is readable. In addition to language, paid apps were also excluded, This was done to accommodate lower- and middle-income status of users and to also search for accessible and cost-effective resources. Lastly, apps that were developed for a specific event or conference were also excluded as they are found to be non-functioning outside of event purposes and thus unusable by the adolescent population.

**Figure 1:**
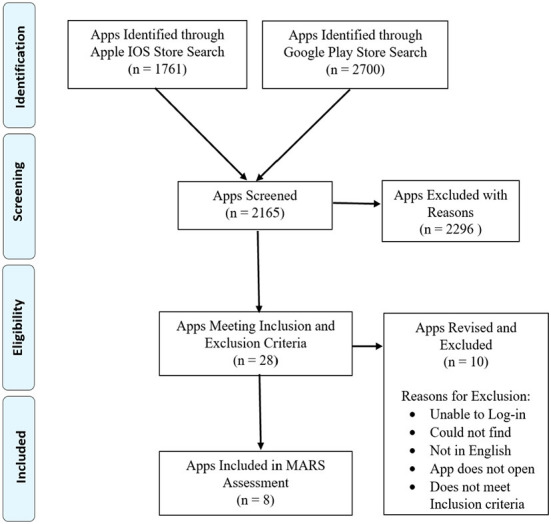
Cumulative scores for each app's MARS subdomain.

**Figure 2:**
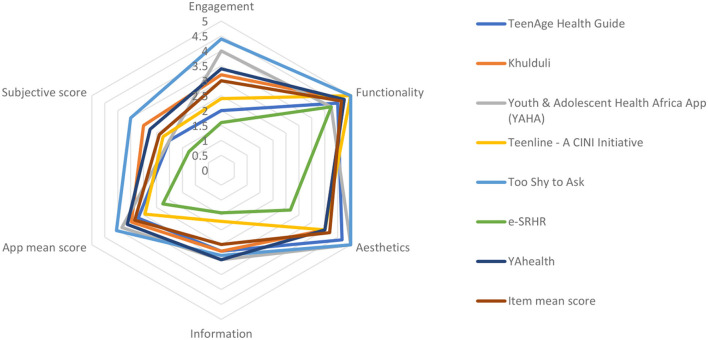
MARS mean item scores for each app.

After screening, the included apps are analyzed using the MARS. Data extracted from the MARS was then converted to a graph (Figures 1, 2). Lastly, we evaluated for app quality and any areas of improvement by comparing item mean scores to each SRH app specific to adolescents in LMICs.

### MARS quality assessment

The MARS is a validated tool used to evaluate the quality of mobile health applications (31). It is a simple and reliable tool that not only classifies and assesses the quality of mobile health apps but also can be used to provide evidence to app developers on creating new higher quality apps (31). Furthermore, a 2020 study found that the MARS tool has good to excellent reliability and objectivity (32). The MARS has a high level of interrater reliability that is ensured by using multiple raters who have been either trained or developed a shared understanding of the target group of apps (31). In this study, two reviewers were trained on how to use the MARS and assess apps. When ambiguity arose, both reviewers clarified the meaning of MARS items and reviewed until a consensus was reached. This approach was done rather than averaging the scores of both reviewers to maintain a high level of interrater reliability. Objectivity was ensured by excluding the subjective quality subscale from the overall mean app quality score (31). Due to its high interrater reliability and objectivity along with its simple and multidimensional uses, the MARS tool was used to assess the quality of mobile apps in this study.

The MARS tool consists of five subscales: engagement, functionality, aesthetics, information, and subjective quality. Within each subscale are three to seven items. For instance, the functionality subscale contains three items: layout, graphics, and visual appearance. Layout item examines whether the app's arrangement and size of buttons, icons, and content are appropriate and zoomable if needed. The second item, graphics, asks how high the quality or resolution of the buttons, icons, and content are. Lastly, visual appearance examines the quality of appearance of the app, and how memorable or poorly designed the smartphone application is. Each item consists of a scale of one to five. Reviewers will assign each item a score from this scale and then later assign a consensus score. These scores are then averaged so that each app can be ranked and compared in terms of app quality (31).

### Data extraction

To analyze apps using the MARS scores, data must first be extracted. We used the MARS scale, a scale-based expert assessment tool, to characterize smartphone applications. Using Excel, we categorized our included apps in a tabular format and two reviewers (AP, SK) independently scored each app to collect our data. To assess each app using the MARS tool, two reviewers (AP, SK) installed each approved Apple IOS app and Android Play store app onto their respective devices. Once installed, reviewers created an account for the apps so that content could be accessed. Then, reviewers spent 10 mins interacting with each app before using the MARS scale to calculate item scores. Reviewers first filled in descriptive and technical information about the app. They then proceeded to app quality rating. Reviewers start with the engagement subscale, for each item – entertainment, interest, customization, interactivity, and target group – and assigned a score out of five. The same was done for the rest of the subscales (functionality, aesthetic, information, and subjective quality) and items. When reviewers began rating the information subscale, published literature was searched to examine whether apps had been trialed or tested. Apps that had not been tested at all were assigned a score of zero rather than N/A so that results could be quantified and converted to a graph. Data were extracted using this method for each of the 8 included apps. In the end, a consensus score was agreed upon by the two reviewers (AP, SK) and a final item score was assigned to maintain the MARS level of high interrater reliability.

## Analysis

After extracting the data, we used descriptive statistics to analyze our data. We calculated the scores for each subscale item and then averaged the items to achieve a mean subscale score. These scores were then converted to a score out of five using a 5-point Likert-type scale, thus creating a total or overall app quality score out of 5 A score out of 5 is established so that it can be easily interpreted like the overall star ratings found in app descriptions. This was done because there is a high correlation between the MARS quality total score and its overall star rating therefore indicating that the overall MARS quality scores can capture the perceived overall quality described by the star rating (31). Another reason this process was done is because the MARS score and its subscale scores consist of high interrater reliability and internal consistency (31). To ensure objectivity, we excluded the subjective quality score from the total MARS mean scores. This is because the subjective nature of the app can decrease the objectivity of the MARS total score (31).

Using the MARS mean total scores, subscale scores, and item scores, we were able to rank order each app. MARS mean scores describe the overall quality of the app while the subscale and item scores describe the app's specific strengths and weaknesses (31). Therefore, these scores were used to identify any gaps in app quality and any areas of improvement.

## Results

### Screening findings

Our search strategy retrieved a total of 1,059 mobile applications for the Apple IOS store and 1,106 mobile applications for the Google play store. From the Apple store, 702 applications were duplicated and removed. While 1,594 applications from the Play store searches were duplicates and removed. Out of the remaining applications, 28 total applications were reviewed against the a-priori inclusion criteria, 20 applications were revised and excluded on this initial screening and 8 moved forward to the MARS assessment. Of the 28 mobile apps, all 6 mobile apps found in the Apple store were revised and excluded from the MARS assessment while only 14 of the 22 Play store apps were revised and excluded. The final 8 apps assessed using the MARS were all from the Google Play store.

Common reasons for the exclusion of 20 apps during the screening include reviewers unable to log in to the app, the app no longer available on the App store or play store, app content did not contain English, and the app no longer met inclusion criteria. For example, Too Shy to Ask, was available on both the Apple and Google platforms. However, it is important to note that this app could not be found in the Apple IOS store when searching for it in the search bar. One of the apps did not target adolescents living in LMICs and another required payment to access SRH content and resources.

After screening, apps that were included in the MARS assessment are: Digital Platform for Adolescent Health, TeenAge Health Guide, Khulduli, Youth and Adolescent Health Africa App (YAHA), Teenline - A CINI Initiative, Too Shy to Ask, e-SRHR, and YAhealth. These apps were then classified based on platform, focus, theoretical background, affiliations, age group, technical aspects, and region of development. All these eight apps functioned on the Android platform only. Content found in the apps mainly focused on topics such as alcohol/substance use, anxiety/stress, depression, and physical health. There were two apps, e-SRHR and YA health which incorporated information on relationship issues. Whereas, another two, Digital Platform for Adolescents and Too Shy to Ask, included content on behavior changes. The MARS also asks reviewers to classify apps based on theoretical background or strategies developers incorporated in the apps. All eight apps provided some form of advice/tips/strategies or skills training to adolescents. They also offered forms of ASRH information or education in their apps. Some apps, Digital Platform for Adolescents, Too Shy to Ask, and Khulduli provided feedback. For instance, both apps contained SRH content quizzes in which the user can receive feedback on if they answered correctly or incorrectly. Lastly, two apps, Khulduli and YAhealth, allowed monitoring and tracking. More specifically, both apps allowed users to track their menstruation cycle. In addition to background, apps were also classified based on affiliation. Digital Platform for Adolescents, Khulduli, and YAhealth were affiliated with government programs. Teenline – A CINI Initiative and Too Shy to Ask were developed in collaboration with the NGOs. Lastly, TeenAge Health guide was developed by a university, Lady Hardinge Medical College. Of the eight apps, TeenAge Health Guide targeted both adolescents and the general population. While, Too Shy to Ask targeted adolescents, young adults, and adults as well. The rest of the six apps only focused on adolescent and young adult populations. With regards to technical aspects, two out of eight of the apps, TeenAge Health Guide and e-SRHR, could not be assessed as either contained features found in the 6 categories described in the MARS. Three out of the eight apps, Digital Platform for Adolescents, Youth & Adolescent Health Africa app, and Too Shy to Ask, featured an app community in which adolescents can ask and respond to others' questions or comments. Two of the apps, Youth & Adolescent Health Africa App and Khulduli, required web access required to access additional functions of the apps. One app, Too Shy to Ask, provided password protection upon entering the app and also required login feature to access app contents. In terms of region of development, Youth & Adolescent Health Africa App (YAHA) and e-SRHR were developed in Uganda. Furthermore, TeenAge Health Guide and Teenline - A CINI Initiative was developed in India. In addition to Uganda and India, the app Khulduli was developed in Nepal. An LMIC that developed the Digital Platform for Adolescents is Bangladesh. Lastly, it is unknown what country is responsible for developing apps Too Shy to Ask and YA health.

### Study findings

#### Results of MARS subdomains and items, and app-related strengths and weaknesses

The 8 included mobile applications have an overall mean app quality score of 3.3/5. These apps scored highest in the functionality subdomain with a mean score of 4.6. Whereas, information scored lowest as part of the overall mean app quality, at 2.5. Overall subjective quality score rated low at 2.4. The rest of the subdomain mean scores are summarized below in Table 1.

**Table 1:** MARS subdomain scores and overall app quality scores.

**App name**	**Engagement mean score**	**Functionality mean score**	**Aesthetics mean score**	**Information mean score**	**Subjective quality mean score**	**App quality score**
Digital platform for adolescents	4	4.5	2.67	2.57	3.25	3.40
Teen age health guide	2	4.5	4.67	2.71	2	3.21
Khulduli	3.2	4.75	4	2.71	3	3.47
Youth & adolescent health Africa (YAHA)	4	4.25	5	3	2	3.84
Teenline – A CINI initiative	2.4	5	4	1.71	2.25	2.95
Too shy to ask	4.4	5	5	2.85	3.5	4.05
e-SRHR	1.6	4.25	2.67	1.43	1.25	2.26
YAhealth	3.4	4.75	4	3	2.75	3.63

#### MARS subdomains

**Figure 3:**
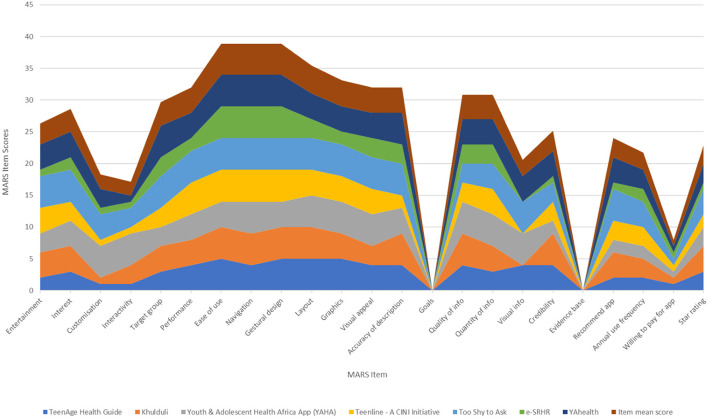
PRISMA diagram of the app screening process.

As seen in Figure 3, the individual app means scores range from 2.26/5 to 4.05/5. From highest to lowest the apps score: Too Shy to Ask (4.1/ 5), Youth and Adolescent Health Africa App (YAHA) (3.8 /5), YAhealth (3.6 /5), Khulduli (3.0 /5), TeenAge Health Guide (3.2/ 5), Teenline - A CINI Initiative (2.9/ 5), e-SRHR (2.3/ 5). Too Shy to Ask scored highest in the Functionality and Aesthetics at 5/5, and Engagement, at 4.4/ 5, subdomains. However, for the Information subdomain, YAhealth and Youth & Adolescent Health Africa App (YAHA) score highest at a tied score of 3/ 5.

#### Apps strengths

The eight mobile apps assessed using the MARS tool highlighted that the apps scored highest for ease of use, navigation, and gestural design (Figure 1). Ease of use describes, “How easy is it to learn how to use the app; how clear are the menu labels/icons and instructions?” While, navigation inquires, “Is moving between screens logical/accurate/appropriate/ uninterrupted; are all necessary screen links present?” Gestural design, “Are interactions (taps/ swipes/ pinches/ scrolls) consistent and intuitive across all components/screens?” scored high. Following gestural design, performance scored next. These results suggest that mobile developers and researchers have ensured adequate functionality of the apps and should continue to include them in future app development.

Other mean items that scored adequately are layout, graphics, and visual appeal of the aesthetics subdomain. Layout asks reviewers if the “arrangement and size of buttons/icons/menus/content on the screen is appropriate or zoomable if needed?” The mean item score of graphics is slightly lower than the layout and it investigates the quality of graphics and visual design used within the app. Visual appeal scored slightly lower than graphics. This MARS item describes the level of attractiveness and appeal of the app through graphics and colour to enhance app features. These findings highlight that aesthetics, second priority to functionality, was satisfactorily developed by researchers when designing apps targeted to adolescents.

Lastly, the MARS item, accuracy, of the information subdomain scored adequately as well. This item examines whether the app description matches what is displayed within the app. This data implies that developers were somewhat transparent with their users as many of the apps contained all or most of the functions/components described in the app store description.

#### Apps weaknesses

Our study identified two major gaps in the MARS item scores (Figure 1). The first gap was the goal item score. This item asked the question, “Does the app have specific, measurable, and achievable goals (specified in the app store description or within the app itself)?” For all eight apps, the score for goals was zero. The second gap was the evidence base. This MARS item score was also zero for all apps. Evidence base asks whether or not the app has been tested and contains verifiable evidence published in scientific literature. Before rating the information subdomain, reviewers carried out a literature search in which no results appeared. Hence, a score of zero was assigned to all apps. These gaps highlight the need for researchers to incorporate goals and a strong evidence base for the future development of SRH mobile apps to meet the needs of its users better.

In addition to goals and evidence base, there was a significant decrease in scores for the following items: willingness to pay, interactivity, and customization. Willingness to pay scored the least among the three, and it entails whether the user would pay for this app or not. Interactivity, “Does it allow user input, provide feedback, contain prompts (reminders, sharing options, notifications, etc.)? Note: these functions need to be customizable and not overwhelming in order to be perfect,” scored slightly above willingness to pay. Customization scores slightly above interactivity and it asks, “Does it provide/retain all necessary settings/preferences for app features (e.g., sound, content, notifications, etc.)?” These results suggest that following evidence base and goals, researchers should then consider willingness to pay and focus on strengthening customization and interactivity so that their target audience can be more engaged.

## Discussion

This environmental scan synthesized all existing SRH mobile apps targeted to adolescents in LMICs and evaluated their quality and characteristics using the MARS tool. Despite the growing development of mobile apps, little research has been done on evaluating SRH apps targeted to adolescents in LMICs. This environmental scan aims to fill that gap and provide a comprehensive overview and assess the characteristics and quality of these ASRH apps for those living in LMICs. The findings from our environmental scan add to the issue of whether mhealth interventions can provide accessible and evidence-based knowledge to adolescents living in LMICs. The increasing availability of mobile phones presents exciting opportunities in overcoming barriers faced by adolescents in developing countries (9). These obstacles include confidentiality, access to cost-free services, accessibility to stigma-free education, and restrictive SRH laws to name a few (7, 9).

Despite the eight apps included in this review, there remain few apps developed specifically for SRH needs of adolescents in LMICs. The lack of effective ASRH apps necessitates further app development since many common avenues of providing comprehensive SRH education have been ineffective against the barriers found in LMICs (33). Political and social factors can also heavily influence prioritization of ASRH services in LMICs, despite the economic and scientific evidence provided (34). In terms of social elements, school-based programs were found to be the most common approach for SRH education in Sub-Saharan Africa, however, their effectiveness is yet to be evaluated (35). Furthermore, community-based programs focused on HIV prevention rather than providing comprehensive SRH education to adolescents due to ideological and financial restrictions in developing countries (33). Another avenue for receiving ASRH education in LMICs is through parents (33). However, researchers noted that many parents were not taught SRH education themselves or felt discomfort thereby impeding their ability to guide their children (33). Furthermore, many LMICs have differing cultures, social attitudes, and policies regarding ASRH. For example, community members in rural Kenya perceive adolescents carrying condoms as deviant and should be subjected to punishments such as beatings (36). In fact, Western Kenya contains education policies that sanctions adolescents found to be carrying condoms in school (36). In terms of politics, the majority of restrictive abortion laws are found in LMICs (37). Moreover, many LMICs that still contain a low legal age of marriage for girls which can potentially lead to increased adolescent pregnancies and maternal death (38). To deal with these barriers, government commitments and mobile phone technologies can potentially lead to positive results (34, 39). In a report describing political commitments that can improve ASRH, researchers report that a government's commitment to integrating adolescent-friendly services into public health policy, such as HIV/AIDs prevention programs, has had positive outcomes on progressing ASRH and making it a national concern (34). Mobile phone technologies, such as apps, can be particularly valuable for adolescents in LMICs as information regarding contraception can be provided discretely and conveniently (39). These studies highlight the need for comprehensive ASRH education that is not only free from consequences but is also tailored to the needs of adolescents in these specific social and cultural contexts. Even in the eight apps that have been developed for these purposes and within the LMICs context, our review highlights that more work is needed in the development of apps that are useful and usable for this population.

Our study identified eight SRH mobile apps developed for adolescents in LMICs that were scored using the MARS scale. Although the MARS scale does not test for cultural context suitability, reviewers noted that some apps did include specific information relating to the app's origin of development. For instance, Too Shy to Ask was developed by the WE foundation and India's Metropolis Health solutions and contained information on the legal rights of Indian women and case studies examining Indian adolescents. Furthermore, this information was available not only in English but also in Hindi, the language of the origin of development.

The MARS tool showed us that all apps had the highest scores in the Functionality subscale (4.6) and lowest scores in the Information (2.5) and Subjective Quality (2.4) subscales. A higher rating for functionality demonstrates that app developers prioritized performance, ease of use, navigation, and gestural design over other items such as those found in the information and subjective quality subdomains. Despite a high scoring of items found within the functionality subscale, there exist two major items from the information domain in need of development: evidence base and goals.

Although mHealth interventions can provide low-cost services, they often lack delivering evidence-based information (40). This was evident in our study, where there is a sharp drop to zero for evidence base (see Figure 1). This finding is consistent with other studies since many users have expressed scepticism and are reluctant to rely on mHealth tools because of their lack of credibility and validity (40). Adolescents specifically have expressed concern over the accuracy of health-related content found online and are reported to be drawn to information from reliable sources such as healthcare professionals or experts (41). These findings suggest that researchers should highly consider prioritizing evidence-based information when developing mobile apps for adolescents.

In addition to evidence, goals were also significantly absent across all apps. Literature reveals that setting specific and attainable goals can improve adolescent cognitive and social development (42). The process of goal setting itself builds self-regulation as it requires individuals to identify a goal, take necessary steps, monitor their performance, and evaluate and adjust their strategies (43). Researchers of previous studies have reported that participants found goal setting and goal attainment features as necessary motivational factors that should be included in mobile apps (44). Furthermore, they described goal setting as an important contributor to maintaining self-discipline, gradually changing their behaviors, and allowing them to monitor their progress and receive real-time feedback (45). By empowering adolescents to set goals via mobile apps, developers can create higher quality apps.

Researchers should also focus their attention on interactivity and customization when developing apps for ASRH purposes in LMICs. The MARS tool has shown that interactivity and customization items are significantly lacking in mobile apps (Figure 1). Interactivity is an essential component of app development as they not only increase user commitment and learning but also brings about a sense of belonging to the users (46). Previous research on mobile apps for adolescents reported that interactive components of an app improved adherence, involvement, and motivation (17). Another weakness found in many apps is customization. This component allows users to feel autonomous and thus more engaged with their health (47). In many LMICs, adolescents face numerous barriers in accessing SRH information and may potentially feel a lack of control over their health. A study on perceptions of ASRH and rights reported that adolescents lack the autonomy to access SRH information and services due to the taboo and stigma associated with this subject (48). By strengthening customization and interactivity features within mobile apps, researchers can design more effective apps for adolescents in LMICs.

A probable reason for a low willingness to pay score is because of low ratings found in other items across the MARS scale. An improvement in customization, interactivity, evidence base information and goal-setting features can positively influence user willingness to pay for these ASRH apps. Based on our findings, we suggest program developers and researchers strive to implement evidence base and goal-setting components and strengthen customization and interactivity components when developing future mobile app interventions for ASRH purposes.

### Limitations and future research

Although the MARS tool has high interrater reliability, reviewer differences in education and culture can impact the scores given. This is because differences in opinions and how different cultures might teach ASRH education can influence the reviewer's subjective quality scores. Therefore, the findings should be interpreted considering several limitations.

Before rating mobile apps using the MARS, reviewers spent 10 min engaging with each app. This short duration gives rise to potential errors in interpreting app information or unintentionally excluding content necessary for rating apps. Moreover, having two reviewers assess the same apps regardless of needing a consensus score, can also lead to potential exclusions and mistakes.

Since app analysis is done from a Canadian adult perspective, the research team's positionality vs. the culture of the populations that these apps are meant to serve can potentially result in a higher score. In particular, all members of the research team have multiple post-secondary degrees and are residents of a higher-income country, Canada. This can influence the subjective quality subscale as researchers are living in an environment with greater resources, and thus may expect higher standards. For example, the ease-of-use MARS item asks reviewers how easy it is to learn to use the app. When exposed to more resources in higher-income countries, researchers may have a greater technology literacy and competency that may result in higher-than-normal scores rated. For a more accurate evaluation of ASRH apps for LMICs, consultation with researchers residing in these areas should be undertaken.

A unique feature found in many ASRH mobile apps was the clinic or nearby services' locative information. App reviewers were unable to evaluate this feature as it was unavailable in their region. If this app were evaluated in the region the app is meant to serve, a higher score may be awarded.

Furthermore, we did not include apps in languages other than English in our study due to feasibility reasons. This may have limited the number of apps included in this review given that many LMICs have a primary language other than English. In addition to languages, paid apps were excluded from our paper. As our target population is adolescents from countries of lower- and middle-income, many may be unwilling or unable to spend money on apps. For this reason, we chose to exclude paid apps. A potential limitation of excluding paid apps is the exclusion of higher quality apps that target adolescents in LMICs from this study.

Additionally, we cannot assess connectivity and internet quality in the regions where these apps are being accessed. We hope that most of the apps' content can be accessed without the need for a high-speed or quality internet connection. However, we did not include this in our inclusion-exclusion criteria as this was difficult to screen for. This issue can potentially impact whether app information and services reach the end user.

Lastly, as this study is providing an overview of the quality and characteristics of SRH mobile apps designed for adolescents in LMICs, it is important to consider whether cultural context suitability is included within the apps. However, the MARS tool is not designed to assess whether apps contain culturally acceptable information. Moreover, the closest information relating to culture the MARS collects is information on who the app developer is and whether the quality of information is accurate, clearly communicated, and relevant to the topic of the app (31). Further assessment is necessary to evaluate accurate and culturally acceptable information on the effectiveness and usability of mobile apps.

As digital health tools are rapidly updating, further investigation is needed to analyse the efficacy and usability of these apps. Ultimately, additional research is necessary to understand the differences in the teaching of ASRH among different cultures to appropriately evaluate these mobile apps for LMICs.

## Conclusion

Mobile health interventions in the form of apps have immense potential in providing accessible, confidential, and stigma-free SRH services and information to adolescents in LMICs. Despite many apps available claiming to provide these services, very few contain evidence-based and goal-setting ASRH information. This environmental scan thoroughly classifies and rates the quality of current SRH mobile applications targeted toward adolescents in LMICs, their strengths and weaknesses, and suggestions for future app development.

## Data availability statement

The original contributions presented in the study are included in the article/supplementary material, further inquiries can be directed to the corresponding author/s.

## Author contributions

Initial conception and design: SM. App screening, data analysis, literature search, and manuscript writing: AP. Data extraction: AP, SK, and SL-P. Manuscript revisions: SL-P and SM. Manuscript feedback: ZL. All authors contributed to the article and approved the submitted version.

## Conflict of interest

The authors declare that the research was conducted in the absence of any commercial or financial relationships that could be construed as a potential conflict of interest.

## Publisher's note

All claims expressed in this article are solely those of the authors and do not necessarily represent those of their affiliated organizations, or those of the publisher, the editors and the reviewers. Any product that may be evaluated in this article, or claim that may be made by its manufacturer, is not guaranteed or endorsed by the publisher.
